# Relational Factors and HIV Testing Practices: Qualitative Insights from Urban Refugee Youth in Kampala, Uganda

**DOI:** 10.1007/s10461-021-03567-4

**Published:** 2022-01-31

**Authors:** Carmen H. Logie, Moses Okumu, Maya Latif, Samantha Parker, Robert Hakiza, Daniel Kibuuka Musoke, Simon Mwima, Shamilah Batte, Peter Kyambadde

**Affiliations:** 1 Factor-Inwentash Faculty of Social Work, University of Toronto, 246 Bloor Street West, Toronto, ON M5S 1V4 Canada; 2 United Nations University Institute for Water, Environment and Health, Hamilton, ON Canada; 3 Centre for Gender &, Sexual Health Equity, Vancouver, BC Canada; 4 School of Social Work, University of Illinois Urbana-Champaign, Urbana, IL USA; 5 Young African Refugees for Integral Development (YARID), Kampala, Uganda; 6 International Research Consortium, Kampala, Uganda; 7 National AIDS Control Program, Ministry of Health, Kampala, Uganda; 8 OGERA Uganda, Kampala, Uganda; 9 Most at Risk Population Initiative Clinic, Mulago Hospital, Kampala, Uganda

**Keywords:** Refugees, Youth, Uganda, HIV testing, Social support

## Abstract

Despite the global phenomenon of refugee urbanization, little is known of relational contexts that shape HIV testing among urban refugee youth. We explored perspectives, experiences, and preferences for social support in HIV testing among refugee youth aged 16–24 in Kampala, Uganda. We conducted five focus groups with refugee youth (n = 44) and five in-depth key informant interviews. Participant narratives signaled relational contexts shaping HIV testing included informal sources (intimate partners and family members) and formal sources (peer educators and professionals). There was heterogeneity in perspectives based on relationship dynamics. While some felt empowered to test with partners, others feared negative relationship consequences. Participant narratives reflected kinship ties that could facilitate testing with family, while others feared coercion and judgment. Peer support was widely accepted. Professional support was key for HIV testing as well as conflict-related trauma. Findings emphasize bonding and bridging social capital as salient components of enabling HIV testing environments.

## Introduction

There were 82.4 million forcibly displaced persons at the end of 2020 [1], and 70% live in urban regions [2]. With 1.4 million refugees, Uganda is the fourth largest refugee hosting nation in the world and the largest in Sub-Saharan Africa [1]. More than 90,000 of Uganda’s forcibly displaced persons live in the capital city of Kampala, and of these, one-quarter are youth aged 15–24 [3]. Most of Kampala’s refugees live in slums [4–6], environments characterized by poverty, precarious housing, overcrowding, violence, and elevated HIV and sexually transmitted infections (STI) prevalence [7–14]. Forcibly displaced persons may have elevated HIV exposure due to the convergence of social and structural drivers of HIV, including sexual and gender-based violence (SGBV), poverty, and constrained access to sexual and reproductive health (SRH) services [15–18]. Yet systematic reviews note how the needs of refugee youth have been overlooked in SRH programs, as have the needs of urban refugees [15–18]. There is a dearth of HIV testing programs tailored for urban refugee youth.

Notable gaps in HIV testing—a key entry point to HIV prevention and care cascades [19]—exist for urban refugee youth in Kampala. For instance, cross-sectional studies have noted testing uptake of 56% among urban refugee youth living in slums in Kampala [20], far from the UNAIDS goal of 95% of people knowing their status to achieve an AIDS Free Generation [21]. While three-quarters of a sample (n = 445) of urban refugee youth in Kampala knew about HIV testing availability in their community, far less (56%) reported having ever been tested for HIV in their lifetime [20]. In this study, the stigma youth experienced for being sexually active and engaging in SRH care was associated with reduced odds of ever having tested for HIV [20]. Qualitative findings have also pointed to the important role of intersecting forms of stigma, including HIV, adolescent SRH, refugee, and sex work stigma, as well as cost and transportation challenges, in producing HIV testing barriers for urban refugee youth in Kampala [22, 23]. Less is known about relational contexts of HIV testing decision-making among urban refugee youth.

Relational contexts include the larger power structures that shape interactions, as well as the ways that relationships can reproduce or challenge power inequities [24]. Relationality, the interconnectedness between people and places, includes *expressive* domains (e.g. working on goals and priorities with other persons) as well as *instrumental* domains (e.g. sharing resources and skills) [24]. Relational transactions and processes shape identities, wellbeing, and knowledge production. Shifting the focus of behavior change from individual agents to their wider environment can bring into focus the socio-environmental situations that shape daily interactions that enable or constrain decision making [25]. Relational contexts that produce HIV enabling environments have been conceptualized as including social support, social capital, and recognition of rights [26]. Social support is multifaceted and includes different sources (e.g. family, friends, significant others), quality, and quantity [27]. Most social support is generated from informal sources, such as family and friends, and can be supplemented by formal sources of support offered through organizations [28]. Support is also multi-dimensional, comprised of emotional (e.g., caring, empathy), instrumental (e.g., tangible aid), appraisal (e.g., help with self-evaluation), and informational (e.g., advice, information) dimensions [29]. Social capital can include relationships that foster *bonding*, such as between persons from a shared social identity group (e.g. between refugee young women) and/or *bridging*, whereby persons build connections with those with increased access to power (e.g. between refugee young women and government stakeholders) [30, 31]. Persons may participate in both formal social networks, such as religious institutions and HIV support groups, as well as informal social networks in families and neighborhoods, in order to build community connections and enact personal and social change goals [32]. Taken together, these social relations can nurture HIV competent communities [30], whereby persons share information about HIV and support one another to overcome barriers to engaging in HIV prevention and care. This approach moves beyond simply providing information to facilitating discussion of how this information can be applied to real life practices [30].

Health promoting HIV enabling environments can nurture solidarity and resilience, as well as reduce power inequities [26, 33]. Yet challenges for creating HIV competent communities include inequitable social norms and social networks that share misinformation. Relational contexts thus have the potential to shape both HIV risks *and* protective factors in the structuring of social networks, social interactions, and daily lived experiences. There are knowledge gaps regarding characteristics of HIV competent communities from the perspective of urban refugee youth. A broader scan of HIV research literature with non-refugee populations identified relational influences on HIV testing practices. For instance, relationship dynamics with family, peers, and partners may have both negative or positive influences on youth HIV testing practices [34, 35]. For instance, among youth aged 14–24 in Kenya, 22% reported that their decision to test was influenced by a peer, 20% by a partner, and 12% by a parent [36]. Family, peers, and partners might offer support, positively influencing HIV testing among youth [34]. Among orphaned youth in Ethiopia, Kenya, and Tanzania, increased perceived social support was associated with past-year HIV testing [37]. Conversely, fear of family, friends, peers, or partners discovering one’s HIV positive serostatus as well as a perceived lack of support are reported testing barriers among young people in South Africa [38]. Peer-led approaches to increase HIV testing were associated with increased HIV testing among men who have sex with men in a systematic review [39].

Micro-social environments comprise social and physical environments that have the potential to exacerbate or reduce harm through shaping perceptions of health risks and practices, community values and norms, social relationships and networks, and neighborhood cohesion [25]. Urban refugee youth in Kampala’s slums experience micro-social environments of poverty and elevated SGBV exposure [40], and macro-social contexts of intersecting stigma toward refugees, HIV, sexually active adolescents, and gender inequities that may converge to reduce HIV testing uptake [22, 41, 42]. It is thus particularly important to understand relational contexts of HIV testing among this population, as refugees may experience disruptions to social and community networks and family dynamics due to the effects of conflict and displacement [43–46]. O’Laughlin et al.’s thoughtful social ecological framework conceptualizes barriers to HIV clinic attendance among refugee adults living with HIV in Nakivale settlement in Uganda [47]. They found that social support from family and friends helped persons to overcome barriers to HIV care engagement; for instance, material (transport funds, childcare) and emotional (hope, moral) support facilitated care engagement. Yet HIV positive serostatus disclosure was selective, and fear of stigma and discrimination presented disclosure barriers that resulted in social isolation [47]. Their work on HIV testing with refugee adults in Nakivale signals the importance of community sensitization, stigma reduction, and engaging with peer educators in HIV testing interventions [48].

Better understanding the social environments of HIV testing decision-making among urban refugee youth in Kampala can inform tailored testing initiatives that employ a strengths-based focus and leverage urban refugee community solidarity and networks [49]. In response, this manuscript aims to address knowledge gaps regarding the relational factors that shape HIV testing practices with urban refugee youth in Kampala. We explore urban refugee youth perspectives, experiences, and preferences for social support in HIV testing.

## Methods

### Study Design and Data Collection

This qualitative study of refugee youth ages 16 to 24 (n = 44) living in five informal settlements (Nsamyba, Katwe, Rubaga, Kansanga, Kabalagala) in Kampala, Uganda was conducted from February-April 2019. This community-based study was a collaboration with refugee youth serving agencies, Ugandan researchers, the Ugandan Ministry of Health, and academics. Five focus groups, approximately 45-min in duration, were conducted with refugee youth: two with young men (one aged 16–19, one aged 20–24), two with young women (one aged 16–19, one aged 20–24), and one with young women sex workers (aged 16–24). Additionally, we conducted five key informant (KI) interviews, approximately 30 min in duration. The KI interviews were conducted at the KI’s workplaces or at community-based agencies in the settlements, and focus groups were conducted a community-based youth refugee agency and at a humanitarian agency.

Peer navigators (PN) (n = 12), six men and six women, were hired to recruit focus group participants through a combination of snowball sampling methods [50], whereby we invited participants and PN to invite others in their social networks to take part in the study, and targeted sampling [50] in order to reach a predetermined number of participants by age and gender (e.g., 6–10 young women aged 16–19). To participate in the focus groups, participants had to be 16–24 years old, able to provide informed consent, and identify as a refugee, displaced, or asylum-seeking person, or have a parent who identified as refugee or displaced. Peer navigators were refugee youth between the ages of 18 and 24 who lived in the target communities; they supported recruitment through the sharing of study information with their peers and helped to pilot the interview guide that was created in collaboration with community partners. KI were purposively sampled by collaborators due to their experience working with refugee youth through community-based agencies, humanitarian agencies, or government and non-government clinics providing HIV/STI services. KI interviews and focus groups were facilitated by trained local researchers in English, French, Swahili, Kinyarwanda, or Kirundi languages, with the support of a translator, audio recorded, and translated and transcribed verbatim into English. Participants provided verbal informed consent prior to participation, and all participants (focus groups, key informant interviews) received the equivalent to $15 CAD reimbursement for time and travel. Research ethics board approval was obtained from: University of Toronto, Mildmay Uganda, and the Uganda National Council for Science & Technology.

We applied thematic analysis, which is theoretically flexible and includes both inductive and deductive analyses conducted concurrently [51, 52]. We followed thematic analysis steps, including having three authors read and become familiar with the transcripts, code the transcripts (CHL, ML, SP), engage in discussions about codes, produce preliminary themes, and review and reassemble themes [53]. This analysis focused on participant narratives that discussed sources and dimensions of social support important to HIV testing engagement. Interview/focus group guides inquired about the role of peer support, family support, and intimate partner support in HIV testing. As this study was theoretically informed by conceptualizations of HIV enabling environments [26] and HIV competent communities [30], we hypothesized that intimate partners, family, and peer support would be important resources for testing, and these were explored using deductive coding approaches [53]. Inductive coding, exploring data driven codes, identified another source of support (health professionals) salient to participants’ HIV testing decision-making. We produced a thematic map of the findings from both inductive and deductive analyses that together identify varying perspectives on social support sources, and dimensions of social support. Member checking was conducted with collaborators at refugee community agencies and HIV service providers in Kampala, including study co-authors.

## Results

The 44 focus group participants (mean age: 20.3, range: 16–24, standard deviation: 2.2) included men (n = 17, 38.6%) and women (n = 27, 61.4%). Countries of origin included the Democratic Republic of Congo (DRC) (n = 29, 65.9%), Rwanda (n = 11, 25.0%), Burundi (n = 3, 6.8%) and Sudan (n = 1, 2.3%). Most were refugees (42/44, 95.5%), one person was undocumented, and one was seeking asylum. Less than three-quarters (n = 31.44, 70.5%) had ever received an HIV test, and the majority (n = 35/44, 79.5%) were unemployed. The key informants (mean age: 32.9, standard deviation: 4.2) included men (n = 3, 60.0%) and women (n = 2, 40.0%) from Uganda (n = 2, 40.0%), Rwanda (n = 2, 40.0%), and the DRC (n = 1, 20.0%).

**Fig. 1:**
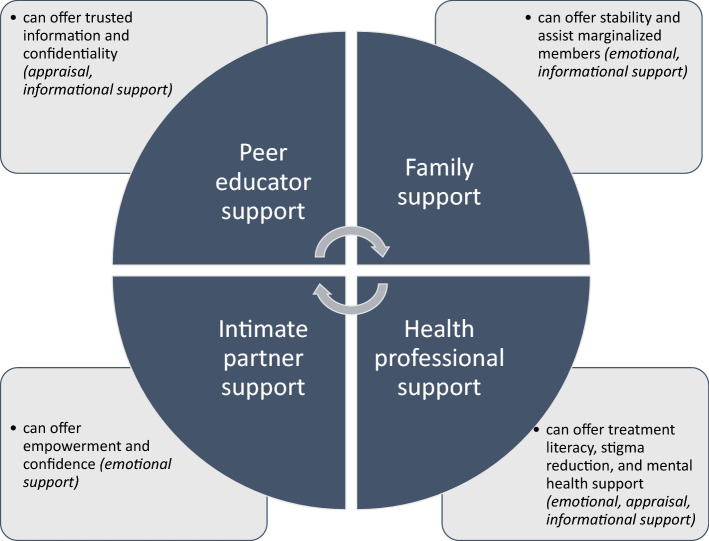
Relational contexts of HIV testing enabling environments among urban refuge youth in Kampala, Uganda

Participant narratives identified informal (intimate partner, family) and formal (peer educator, health professional) sources of social support as salient to HIV testing decision-making. The facets of support that emerged as relevant for testing are presented in Fig. 1.

### Informal Sources of Social Support

#### Intimate Partners

The effects that HIV testing practices could have on relationships with intimate partners was a frequently raised topic amongst participants when making decisions regarding engaging partners in testing. Participant responses were heterogenous and reflected diverse concerns and perspectives on both positive and negative outcomes that testing engagement, and test results, could have on intimate partnerships. Participants described how supportive relationships could encourage HIV testing, discussing how open communication and sharing personal information had the potential to facilitate conditions where both parties could feel secure. “Since you share with your partner everything, deep things,” one young man put forth, “there’s no secrets, and we know that we are safe.” (focus group [FG], young men, aged 20–24).

Women also described how getting tested themselves could help them to feel more confident discussing and enforcing HIV testing with male partners. As one young woman elaborated:

For me it would be very simple. If I have been through counseling and they give it [a test] to me, I would tell my boyfriend that, you know, nowadays things have improved, instead of going to the hospital we can do these things [get tested] together, you can even do it on yourself so that you can know your status whether you are negative or positive. If he refuses it would be up to him, but for me I would have done my job. (FG, young women, aged 16-19)

Another participant similarly reflected on the importance of knowing one’s status and the growing empowerment of women:

It is difficult for them to share with you their status because they have very many partners, but once you know about your own status first of all, and you have that confidence, [then] knowing about your partner’s status will be a must. Because yourself you’re okay, then how come you accept to die just for free? … You have to know also, so women are becoming more powerful. (FG, young women, aged 16-19)

Conversely, many participants felt that engaging intimate partners in HIV testing practices was a more complicated matter—one that could result in weakened trust, conflict, violence, and the dissolution of the relationship. Issues of trust were understood to be pivotal in testing decisions involving intimate partners. “When you tell someone sometimes that you go and test or something like that,” one adolescent young woman explained, “[they] will start thinking that maybe you don’t trust me, things like that, so you can bring that (HIV self-test) kit to that one person and they still refuse it and can even bring up some arguments showing that you don’t trust them.” (FG, young women, aged 16–19) These concerns were also raised by an adolescent young man: “For me, I may want to give it to her [my partner] but to her, she may take it like you don’t trust her.” (FG, young men, aged 16–19) “It might spoil the relationship,” one youth told us, “because there are some girls who are complicated the moment you ask her to test, feel like you don’t trust her. Therefore, they will break the relationship.” (FG, young men, aged 16–19).

The potential empowerment that testing could provide for women as described above was understood as conditional, as it was also dependent on receiving negative test results. Participants described fears that they would be rejected or left by their partner should they test positive for HIV. This was also illustrated by a key informant:

If the girl is already infected, or if she suspects that she has HIV, she will be afraid of being asked for her results. There is also denial, the fear of being neglected, the fear of losing the boyfriend. She may not be willing to disclose her status immediately. However, if she is negative, it will be very, very easy to give it to the boyfriend. She might even force him to take it. (key informant, AIDS service organization)

Another key informant working with sex workers reinforced this concern:

It is hard, and of course you know that once this partner gets to find out if I test positive of course this partner is going to run away and maybe you need that partner around, so it is always more comfortable if you do it [HIV testing] alone but not with your partner. (key informant, sex worker)

Taken together, participant responses signal how supportive intimate partner relationships can encourage and even be further strengthened by HIV testing. However, this outcome was contingent on the stability of relationship, and on the results of the test. When intimate relationships were already precarious, positive HIV test results were understood to have the potential to end relationships.

#### Family

Perspectives on engaging family members in HIV testing decision-making reflected a high degree of ambivalence. Many participants viewed family engagement as important in HIV self-testing, as family members were trusted, understood them, and could care for youth with an HIV-positive diagnosis. The strength of kinship ties emerged as important in HIV testing decisions, evidenced when participants described a family member would be the preferred person to provide them with an HIV self-test. For instance, when asked who they would prefer receiving an HIV self-test kit from—a friend, a peer, or a family member—one young man responded: “the family member, because you won’t neglect blood. The brother, what he’s telling you…you will take it with value.” (FG, young men, aged 20–24) This sentiment was corroborated by another participant, framing support from families as long-term: “A family member, because, if you get any problem, they won’t give up on you so quickly.” (FG, young men, aged 20–24).

In addition to offering stability, family members were perceived to have increased access to other family members compared to outsiders. As a participant explained, they preferred HIV testing with “the member of the family because where the leader or peer educator can’t reach, the member of the family may take charge.” (FG, young man, aged 20–24) The family was further understood as a network for sharing HIV knowledge. Another young man added how family members could support others with less education or information: “some people didn’t go to school, but in the family we understand each other. So, when you have a family member, [they] will explain to you how to use it [HIV self-test kit].” (FG, young man, aged 20–24) Kinship bonds were thus understood as having the potential to facilitate HIV testing in situations of social and educational marginalization.

In contrast, other participants discussed wariness engaging with family members in HIV testing decision-making due to fears of judgment and even potential coercion. To illustrate, a key informant discussed concerns of coercion by family members for HIV self-testing and other prevention techniques:

If you give it [HIV self-test kit] to a family member, they will say, ‘my son, you didn't spend the night here, come and test because I am your mother, so I force you to test.’ Yet testing for HIV is not a must, it should be voluntary. This can cause problems in the family. That is the same issue that happened with condom distribution, when we were taking them to people's homes and they were saying that we are promoting adultery. (key informant, humanitarian agency)

Participants feared judgment for being sexually active if family members knew they were testing for HIV. As a participant described, testing with “the family member it is a big problem because if I tell my auntie to give me [an HIV self-test] and I test myself, that auntie of mine will start asking why this girl is asking for the test kit to test herself, meaning she is suspecting herself.” (FG, young women, aged 20–24) Similarly, another participant noted that “for the family member there is a challenge because it may cause some misunderstanding, and for parents they might think the child is spoilt.” (FG, young women, aged 20–24).

Additionally, concerns were raised that sometimes family members did not disclose their child’s HIV positive serostatus to them due to HIV stigma and/or misinformation. As one key informant related:

You have to engage the parents to understand the benefits of using the HIV self-test. Why? We have had scenarios where an adolescent turns (HIV) positive and the parents say, ‘don't start ARVs [antiretroviral therapy], because those drugs will just weaken you.’ Of course, this is not right but you can't handle this young refugee alone leaving the parents or caretaker out of the picture.” (key informant, AIDS service organization)

These concerns were further corroborated by a service provider who reported:

I do home-based testing where we go to homes and we identify those in care, call them and go and test the family members. That is a bit complicated because most of them don’t want us to go and test the family members. There are those who refuse us to test the children and even that age is very tricky. If they test themselves and can’t read, they will end up asking someone else. There are clients at that age 15-25 years who are taking ARVs every day and they are not aware that these are ARVs. The parents have failed to tell them that they are HIV positive. They tell them the medicine is for liver problems and things like that. You find parents who don’t want their children to know that they are HIV positive. (key informant, hospital-based HIV care)

Thus, although some families were understood as being able to support HIV testing and engage other family members, there were also circumstances where family members could hinder those same efforts and HIV treatment engagement due to larger contexts of stigma and misinformation.

### Formal Sources of Social Support

#### Peer Educators

Where participants were largely unified was in expressing the desire to receive HIV testing information and support from peers, understood as other refugee youth who were trained in HIV prevention. Participants discussed that the benefits of engaging such peers, as distinct from friends, in HIV testing included less judgment, increased confidentiality, and they understood peers to be more knowledgeable. As one young woman told us: “I would go with the peer educator mostly because they have experience, they are more mature than you, there is that comfort they can give to you, the direction, the instructions. Even when you find yourself positive you may not worry so much.” (FG, young women, aged 20–24) Others described the benefit of engaging with trained peer educators who could also support engagement with other sexual and reproductive healthcare needs: “The peer educator is educated about confidentiality [and about] counselling for both (HIV) positive and negative. If we test and we are negative the next thing is, remember we are fighting against HIV but we also can look at issues like unwanted pregnancies, STIs.” (key informant, humanitarian agency).

Women engaged in sex work also reported preferences for peer engagement with HIV testing. As one key informant working at a sex worker agency described:

We have peers operating at different [sex worker] hotspots, so these peers one of their responsibilities is ensuring that they mobilize our members, so when we take out the HIV counselling and testing, the outreaches and all that, it is the peers that are responsible to do the mobilization and communication. I think a peer would be the best option because as you know when it comes to us, sometimes our families have abandoned us because of the work we do, so it will not be comfortable for me to confide in my family or a friend because it is hard for one to disclose even when they find out that you are [HIV] positive. You know a friend would judge you and your family would say that you see now you had to acquire this because you are a sex worker, we have been telling you this and that, however we feel more comfortable being with a fellow sex worker because we are in the same industry and we understand our issues and we will not be judged. (key informant, sex worker agency).

Peer support in this narrative was understood as a way to circumnavigate HIV stigma as well as other forms of intersecting stigma, such as sex work stigma.

#### Health Professionals

Finally, many participants suggested accessing professional health worker support in HIV testing. This was particularly pronounced for wanting counselling alongside HIV testing: “The health workers and the counsellors before they test you, they first take you through some counselling,” one young man noted. (FG, young men, aged 16–19) As another young man told us, “It’s better to go directly to the doctor because you will receive counselling from the doctor regardless of your (HIV) status results.” (FG, young men, aged 20–24).

A young woman highlighted that the advantages of talking to a professional healthcare provider could extend beyond the biomedical realm, commenting on how counselling can help to reduce HIV stigma: “say the result is (HIV) positive, there is a way the doctor engages with you into a counselling session in order not to feel stigmatized or feel undermined in the society, you start a new process.” (FG, young women, aged 16–19) Counselling can also promote living positively with HIV: “It’s very important because it makes the person aware that there’s life after being sick,” another participant told us, “you find that [you] can live for some time knowing that there’s still hope. Therefore, your mind changes after meeting with a doctor, takes you through counselling, so that way, you can have life.” (FG, young men, aged 16–19) These mental health benefits of being supported by a professional when testing were memorably summed up by one young man who, when discussing the idea of HIV self-testing, told us he preferred to access HIV testing at a local hospital:

Going to hospital direct is still better because [if you are] alone, maybe you are in your room alone, and see that you’re positive, you might get a heart attack and die direct because you never had someone to prepare you like the doctor could. (FG, young men, aged 20-24)

These concerns reflect fears surrounding a lack of support and understanding when receiving an HIV positive diagnosis that could be addressed by professional healthcare workers.

The importance of engaging with health professionals was also commonly articulated as a need for mental health support at large rather than for HIV care alone. A number of participants mentioned the risk of suicide after receiving an HIV positive diagnosis, underscoring the stress involved in testing decisions. As one woman told us, “you might kill yourself so you need someone to keep on counseling you on how to conduct yourself, how to take drugs such that you don’t infect others.” (FG, young women, aged 16–19) “There might be a possibility of suicidal tendencies,” a young man similarly related, “but if you have someone checking on you, guiding and encouraging you every week, you can easily regain your courage.” (FG, young men, aged 16–19).

Pre-existing mental health challenges among refugees intersects with these considerations for professional support during HIV testing. As one service provider told us about their experiences working at an AIDS service organization: “Most refugees have mental health issues so when handling them you have to be extra careful. They expect health workers to give them special attention. As a service provider, you have to keep the waiting time very short.” (key informant, AIDS service organization) Others noted that refugee youth often experience persistent mental health challenges due to trauma related to war, violence, and forced displacement. For instance, a key informant at a humanitarian organization described the lasting effects of experiences of sexual violence during migration, telling us that:

In that rape you will find that SGBV (sexual and gender-based violence) is there, then psychosocial and mental problems also arise from that SGBV, that rape. So, we also have a mental health desk and that is comprised of a psychiatric doctor, a clinical psychologist, we have a psychiatric nurse, we have a psychosocial counsellor.” (KI, humanitarian agency)

Thus, the provision of counselling for HIV testing could help to mitigate barriers of fear and stigma by providing social support and information on living positively, as well as serving an important gateway for accessing other mental health support for pre-existing trauma.

## Discussion

Participant narratives reflect multi-dimensional relational contexts that shape HIV testing decision-making among urban refugee youth in Kampala (see Fig. 1). Findings aligned with our hypothesis that informal (intimate partners, family) and formal (peer educators) support sources play important roles in shaping HIV testing practices. Narratives also identified another formal source of support—health professionals—that can facilitate HIV testing, and wellbeing more generally, with urban refugee youth. Our study contributes to the nascent knowledge base on HIV testing with urban refugees to reveal the ways that collectively these formal and informal sources hold the possibility to provide three key dimensions of support [27, 29]: *emotional* (managing stress in testing processes), *appraisal* (better understanding the need for HIV testing), and *informational* (providing HIV information, treatment literacy) support.

Our findings also reveal important complexity within informal support sources regarding HIV testing. While testing could occur within strong intimate partnerships, it could also negatively impact tenuous partnerships and these harms could be amplified with HIV positive test results. Family contexts could provide stable support and knowledge of how to access marginalized family members, including those with low literacy or language barriers, yet simultaneously, family members could re/produce stigma regarding adolescent sexual practices and HIV. Formal support sources were more commonly discussed as beneficial, signaling their potential in offering bonding (peer educators) and bridging (health professionals) social capital that encourages HIV testing engagement. Findings can inform HIV testing and disclosure interventions that span formal and informal support sources and share both the positive and potentially negative implications of engaging a diversity of support networks.

Findings corroborate prior research on the complexity of HIV testing with intimate partners conducted among non-refugee populations. For instance, a body of research shows that fear of negative repercussions including stigma, violence, and rejection from testing positive present barriers for HIV testing uptake among women [54], including pregnant women [55], and couples [56, 57] in Eastern and Southern African contexts. Yet in supportive relationship contexts, HIV testing and disclosure may facilitate care engagement and strengthen relationships [56]. Findings from life history interviews with non-refugee young adults matched by HIV positive and HIV negative serostatus in Rakai, Uganda demonstrated that the nature and quality of sexual relationships were key factors that differed by HIV serostatus [58]. Increased HIV-related communication, trust, and smaller sexual networks were more likely to be reported among HIV negative participants, emphasizing the need for relational and dyadic approaches to understanding the micro-social contexts of HIV risk perception and testing decision-making [58]. Our findings indicate the need to assess sexual relationship power dynamics [59] among urban refugee youth in decision-making regarding HIV testing and disclosure with intimate partners.

Our finding of diverging perspectives toward family involvement in HIV testing decision-making is congruent with prior research with non-refugee youth. For instance, a study with adolescents in Zambia found that: youth commonly sought advice from family members before testing; conceptions of family broadened beyond parents to include siblings and extended family members; and when a family member was unsupportive of HIV testing, this could discourage engagement in testing and result in seeking support from other sources [60]. The quality of kinship relationships — similar to couple-based HIV prevention [61] — was a key consideration that influenced participant perspectives on engaging family. Families have also been conceptualized as a source of hope for people with HIV, both at diagnosis and over the long-term, suggesting the social environment, and families in particular, are “a regulator of hope” [62](p. 18,802). Among adolescents and young adults in Kenya, parents were the most common support person to influence testing decision-making and to accompany a youth to testing for those aged 14–19 [36]. Our findings suggest the importance of family stability to urban refugee youth in accessing testing support—possibly more important for refugee youth who often experience breakdown of family and community networks in conflict—as well as the reciprocal nature of family support, where youth described providing HIV information to marginalized family members.

Participant narratives overwhelmingly supported engaging peers in HIV testing processes, corroborating a robust evidence base on the importance of peer engagement in youth HIV prevention and care cascades [63, 64]. Systematic reviews report that peer education programs in low and middle-income countries [65] and Sub-Saharan Africa [66] were associated with increased HIV knowledge and condom use, yet did not examine HIV testing. In a study with adolescents and young adults in Kenya, those aged 20–24 were most likely to identify being influenced to engage in HIV testing by partners or peers and to go for testing with peers [36]. O’Laughlin et al. identified the importance of engaging peer educators living with HIV in Nakivale refugee settlement in HIV testing educational campaigns [48]. Future implementation science research can explore successful approaches for engaging peer supporters in HIV testing processes with youth in Sub-Saharan Africa [67], particularly among urban refugee youth.

The present study’s finding that some urban refugee youth wanted professional support and pre-test counselling aligns with prior research with youth in Lesotho [68] and South Africa [38] who reported wanting pre-test counselling, information, and support in case of an HIV positive diagnosis. In another study with youth in Nigeria, most participants preferred testing in a health facility with physician-administered tests, although there was heterogeneity in testing preferences [69]. While limited research has explored preferences for professional support with HIV testing among urban refugees, O’Laughlin et al. explored home-based and clinic-based testing in Nakivale settlement in Uganda [70]. They found that home-based testing participants were more likely to be refugees than clinic-based testers, suggesting that this could be due to language barriers at clinics or due to living further from the clinic and experiencing transit barriers [70]. Building on this, our findings suggest the importance of offering a range of accessible testing options for urban refugee youth and ensuring that counselling services are offered in languages spoken by refugees. Further research can explore the ways that HIV counselling can screen for mental health issues and facilitate linkage to mental health support with urban refugee youth.

There were several study limitations. The focus group study design did not include the opportunity for individual interviews with youth, thus some youth may have not felt as comfortable sharing about HIV testing in group setting. We did not explore experiences of sexually or gender diverse youth, or of transgender women or cisgender men sex workers. The prior linkages to a youth refugee agency among most participants could result in them having increased access to social support and HIV knowledge. As this study focused on urban refugee youth, we did not collect information about youth testing experiences in a refugee settlement, and this would give us insight into what is unique and different based on the social context young refugees live in. We also were unable to differentiate experiences based on country of origin or length of time in Uganda, and those could be linked with access to social support. Finally, we did not explore religious support and other formalized social support networks, and these could be important to HIV testing engagement [71]. Notwithstanding these limitations, our study offers a unique contribution to understanding the emotional, appraisal, and informational support domains offered across varying sources (peer, family, intimate partner, professional). These findings can inform tailored HIV testing interventions that leverage these multi-dimensional forms and sources of support to create enabling environments that include both technical (e.g., information, such as treatment literacy) and transformative communication (e.g., awareness of and confidence to address power inequities, such as young women’s awareness of gendered power inequities and confidence to insist on partner testing) [26].

*Places* as a concept, comprising social processes and practices in a particular location [72, 73], are relational—both shaped by and shaping social networks and interactions across the life-course [24]. The quality of places is formed not only by the people inhabiting them, but also by the nature of their interactions [74]. Among refugee youth participants living in Kampala’s slums, social relationships varied in ways that facilitated or inhibited HIV testing engagement. Slums are spatial entities with shared social and physical environments characterized by poverty and inadequate housing and social services [75]. Contextual characteristics of slums that may influence HIV testing include dense, crowded living spaces that afford limited privacy for self-testing, and access barriers, including a lack of health facilities, poverty, and transport costs [23, 76]. Aligned with discourse on place [24, 72], participant narratives reminded us that places such as slums in Kampala are fluid, shifting, and dynamic, and residents can have distinct experiences. Our findings point to the heterogeneity within a particular place—in this example, among urban refugee youth in Kampala—that can inform diverse approaches to HIV testing to create enabling environments [26]. These shared social entities hold the potential for ‘neighborhood effects’ whereby intervention benefits can be widely dispersed in the community [75, 76]—thus HIV testing interventions focused on refugee youth in slums could potentially benefit others (adults, non-refugees) in these communities.

**Fig. 2:**
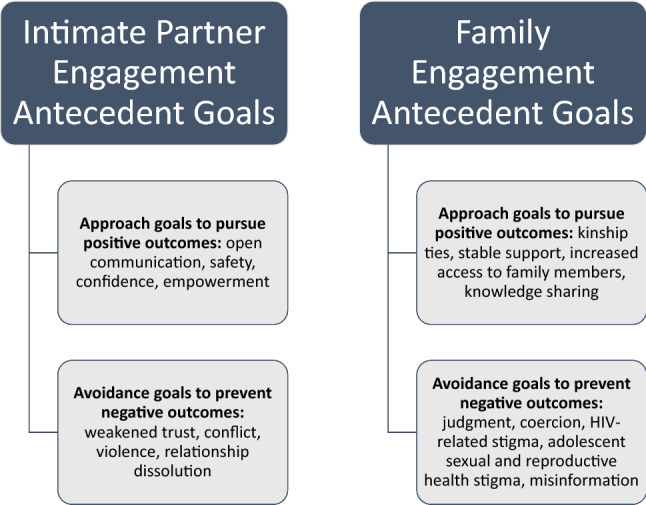
Antecedent goals for engaging informal social support sources in HIV testing decision-making among urban refuge youth in Kampala, Uganda

Our findings offer insight into the importance of offering urban refugee youth professional support for both HIV and mental health needs, signaling the importance of applying a syndemics approach to investigate social contexts that shape co-occurring sexual and mental health disparities among slum-dwelling refugees [18, 77]. Refugee youth narratives also shed light into the potential beneficial and harmful outcomes of engaging informal supports (intimate partners, family) in HIV testing decision-making. We apply the Disclosure Process Model [78, 79] to conceptualize the antecedent goals for engaging these support sources (Fig. 2), with attention to both the approach goals for positive outcomes and avoidance goals for negative outcomes identified in participant narratives. As refugee youth have unique experiences of conflict-related SGBV and family breakdown [80–82], it is particularly important to identify the range of potential outcomes for accessing support in HIV testing to inform decision-making among this population.

Attention to gender dynamics as complex and dynamic is key in HIV testing programs—young refugee women in our study discussed increased women’s empowerment alongside persisting inequitable sexual relationship power. Refugee young women sex workers noted the particular importance of peer support in light of family rejection, signaling the salience of an intersectional approach to gender [83]. Young refugee men appeared to be more in favor of family engagement—they possibly experience less judgment for being sexually active than young women due to inequitable gender norms [84]. Mannell et al. explain that programs need to focus on agency and pleasure rather than risk, and recognize evolving gender norms and relationship dynamics, in order to effectively prevent HIV and intimate partner violence with young women in Southern Africa [85].

Our findings align with the call for HIV testing campaigns to consider HIV status disclosure concerns, risk of partner violence, and social support [54], as well as the shift from a risk framework toward a strengths-based approach that leverages community strengths, resilience, and resources [49]. Building on the concept of HIV competent communities that help persons to acquire information, support and skills to overcome barriers [30], HIV testing interventions with urban refugee youth can facilitate access to bonding (peers, partners) and bridging (family, professionals) social capital. With this approach, HIV testing interventions hold the potential to advance sexual justice—sexual health experiences that are person-centred and advance sexual rights and sex positive practices [86].
